# Growth and Survival Outcomes for Immature Gopher Tortoises in Contrasting Habitats: A Test of Drone‐Based Habitat Assessment

**DOI:** 10.1002/ece3.70509

**Published:** 2024-11-03

**Authors:** Leyna R. Stemle, Julie M. Sorfleet, Chelsea L. Moore, Jack T. Christie, Christopher A. Searcy, Betsie B. Rothermel

**Affiliations:** ^1^ Department of Biology University of Miami Coral Gables Florida USA; ^2^ Archbold Biological Station Venus Florida USA

**Keywords:** chelonian, forage quality, habitat management, juvenile survival, restoration, unmanned aerial system

## Abstract

Juvenile growth rate is a critical demographic parameter, as it shortens the time to maturity and often dictates how long individuals remain vulnerable to predation. However, developing a mechanistic understanding of the factors determining growth rates can be difficult for wild populations. The gopher tortoise (*Gopherus polyphemus*) is an ecosystem engineer threatened by habitat loss and deficient management of pinelands in the southeastern United States. We investigated the factors governing immature gopher tortoise growth and explored the use of drone‐based imagery for habitat assessment by comparing predictive models based on ground‐based plant surveys versus drone‐derived data. From 2021 to 2022, we tracked and measured immature tortoises in native sandhill and human‐modified, ruderal habitat in south‐central Florida. Using quarterly, high‐resolution drone imagery, we quantified plant cover types and vegetation indices at each occupied burrow and measured the frequency of occurrence of forage species by hand. Annual growth rates of immature tortoises in ruderal habitat were higher than those in sandhill and were the highest published for this species. Models based on drone‐derived data were able to explain similar proportions of variation in growth as ground‐collected measures of forage, especially during the late dry season when both types of models were most predictive. Habitat differences in forage nitrogen content were also more pronounced during this season, when dominant ground cover in ruderal habitat (bahiagrass) had much higher nitrogen content than dominant ground cover in sandhill (wiregrass). Despite concerns about potential growth‐survival trade‐offs, tortoises in ruderal habitat did not exhibit lower apparent survival. Our findings indicate that habitat dominated by nutritious non‐native grass can provide a valuable supplement to native sandhill through the mechanism of increased growth rates due to higher forage quality. Finally, our study demonstrates that drone technology may facilitate management by providing less labor‐intensive ways to assess habitat quality for this and other imperiled herbivores.

## Graphical abstract

The gopher tortoise is an ecosystem engineer threatened by loss and deficient management of its pineland habitats in the southeastern United States. To aid management, we investigated the factors governing growth of immature gopher tortoises and explored use of drone‐based imagery as a habitat assessment tool. We used quarterly, high‐resolution drone imagery to quantify plant cover types and vegetation indices at each occupied burrow and measured the frequency of occurrence of forage species by hand. Annual growth rates of immature tortoises in ruderal, grass‐dominated habitat were higher than those in shrub‐dominated sandhill and were the highest published for this species. Models based on drone‐derived data were able to explain similar proportions of variation in growth as those based on ground‐based measures of forage, especially for data acquired during the late dry season when both types of models were most predictive. Our study demonstrates that drone technology may facilitate management.

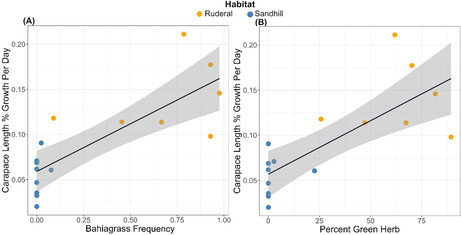

## 1 Introduction

Given the extent of anthropogenic habitat modification, the fate of many threatened species hinges on successful habitat management interventions to stabilize populations and reverse declines (Benayas et al. 2009). The effectiveness of such interventions depends on having an adequate understanding of the relationships between vital rates and habitat conditions—that is, identifying which vital rates and habitat characteristics are the most important targets for management—as well as knowing potential demographic trade‐offs (De Silva and Leimgruber 2019). Entities implementing species management typically lack resources for intensive demographic monitoring and often rely on indicators of habitat quality to gauge whether management is having the desired beneficial effects (Benayas et al. 2009; De Silva and Leimgruber 2019). This issue is particularly acute for long‐lived species, such as many chelonians, owing to the mismatch between the multi‐decadal pace of demographic change and the much shorter timescale of habitat management decisions (Congdon, Dunham, and Van Loben Sels 1993; De Silva and Leimgruber 2019; Folt et al. 2021).

Another common challenge in conserving long‐lived species is the susceptibility of young age classes to predator‐induced mortality (Wilson 1991; Tucker 2000). For example, mortality from depredation is particularly high for turtles during the first few years of life, when the shell has not fully hardened, and predators' gape is not yet a limiting factor (Congdon and Van Loben Sels 1991; Stokes et al. 2006). Therefore, it can be beneficial for many chelonians to maximize growth while in early age classes to reduce the time at a vulnerable size, thus avoiding predation (Congdon and Van Loben Sels 1991; Tucker 2000; Stokes et al. 2006; Mansfield, Wyneken, and Luo 2021). Rapid early growth may also lead to reduced time to maturity and/or larger size at maturity, further enhancing lifetime fitness (Landers, McRae, and Garner 1982; Congdon, Dunham, and Van Loben Sels 1993; Scott 1994; Stamps, Mangel, and Phillips 1998). Specifically, larger body size generally correlates with increased fecundity and survivorship (Messerman et al. 2023; Otten and Refsnider 2024), and reduced age at maturity often allows for more years being reproductively active (Iverson 1992; Congdon, Dunham, and Van Loben Sels 1993; Harris 2014).

While beneficial to later survival, faster growth can be associated with higher mortality in the short term. Growth‐survival trade‐offs occur in diverse taxa, from plants (Inman‐Narahari et al. 2014; Meira‐Neto et al. 2019) to vertebrates (Lindeman 1997; Mangel and Stamps 2001; Wells and Harris 2001). Negative consequences of rapid early growth may include reduced immune function or delayed ossification (Mangel and Stamps 2001) or increased risk of predation while foraging and thus lower survivorship (Mangel and Stamps 2001; Wells and Harris 2001; Brown and Kotler 2004; Quinn et al. 2018). For example, Jefferson salamanders (*Ambystoma jeffersonianum*) prioritize rapid larval growth at the expense of lower survival in the presence of predators (Wells and Harris 2001). In contrast, most young Atlantic salmon (*Salmo salar*) feed during the night, which leads to lower growth rates but also greatly reduces the risk of predation compared to those that feed during the day (Fraser and Metcalfe 1997). As these examples demonstrate, there can be contrasting strategies to optimize fitness in response to unavoidable trade‐offs between early growth and survival.

Growth‐survival trade‐offs have been suggested but not thoroughly investigated in terrestrial turtles (Otten and Refsnider 2024). Young terrestrial turtles may face a trade‐off between forage utilization and predation risk, particularly in open habitats where there is sparse cover to hide from visual predators. This potentially occurs in gopher tortoises (*Gopherus polyphemus*), a threatened, fossorial species restricted to Coastal Plain uplands of the southeastern United States (Pike 2006; Guyer, Johnson, and Hermann 2012). The gopher tortoise is an important focal species for management because of its keystone functions and ecosystem engineering effects (Kinlaw and Grasmueck 2012; Catano and Stout 2015; Figueroa, Lange, and Whitfield 2021). Fire‐maintained pine and sandhill communities on sandy, well‐drained soils provide the highest quality natural habitat for this species (Auffenberg and Franz 1982; Mushinsky et al. 2006). Regular burning is required to maintain the species' preferred open‐canopy conditions (Hermann et al. 2002; Ashton, Engelhardt, and Branciforte 2008), which in turn promotes abundant herbaceous forage and enables faster growth (Mushinsky, Wilson, and McCoy 1994; Hermann et al. 2002). Although early growth is an important driver of demography via effects on age and size at maturity, modeling suggests gopher tortoise population growth rates are most sensitive to annual survival of juveniles and adult females (McKee et al. 2021; Folt et al. 2021, 2022). Hathaway (2012) and Hentges (2014) found that cattle pastures provide abundant, high‐quality forage (resulting in large clutch sizes), but near‐absence of juveniles suggested there was high mortality of eggs or young tortoises in such open habitat. Similarly, while adult gopher tortoises readily use roadsides to forage, anthropogenically created open areas may be ecological traps if there are concurrent risks (e.g., invasive predators and road mortality; Rautsaw et al. 2018). Our knowledge of habitat selection, growth, and survivorship of immature gopher tortoises is relatively limited compared to adults because of the difficulty of observing the small, cryptically colored, and typically less abundant juveniles (Wilson, Mushinsky, and McCoy 1994; Eubanks, Michener, and Guyer 2003; Guyer, Johnson, and Hermann 2012).

Estimates of habitat‐specific growth and survival rates of immature gopher tortoises are needed to guide land management practices and conserve remaining populations. With this in mind, we examined how variation in forage resources affects growth of wild, immature (i.e., juvenile and subadult) gopher tortoises and whether they face trade‐offs between growth and survival in high‐ versus low‐resource environments. Fine‐scale habitat heterogeneity within our study area in south‐central Florida enabled direct comparison of growth and survival between sandhill (low density of mostly native herbaceous plants) and ruderal habitat (high density of mostly non‐native herbaceous plants). A previous study at this site found that adult tortoises residing in ruderal habitat attain larger body sizes (Howell et al. 2020); therefore, we hypothesized that the presence of non‐native herbaceous plants in ruderal habitats provides supplemental forage for young tortoises and facilitates faster growth than experienced by similarly aged tortoises in sandhill habitats. However, we expected there would be lower survival in open, ruderal habitat compared to sandhill, which has more shrub cover. Additionally, we investigated temporal patterns of growth in this subtropical portion of the species' range in relation to seasonal differences in forage abundance and quality.

To enhance the applicability of our findings for species management, we examined how well immature growth rates can be predicted from traditional, ground‐based measures of habitat quality versus data acquired using drones (i.e., unmanned aerial vehicles). Drones are becoming widely used for land management and wildlife conservation (Baratchi et al. 2013; Jiménez López and Mulero‐Pázmány 2019) and may enable more efficient monitoring of habitat quality and species' responses to management (e.g., Scarpa and Piña 2019). Thus, we explored the utility of drones for assessing differences in forage resources for this terrestrial, herbivorous reptile.

## 2 Materials and Methods

### 2.1 Study Site

**FIGURE 1:**
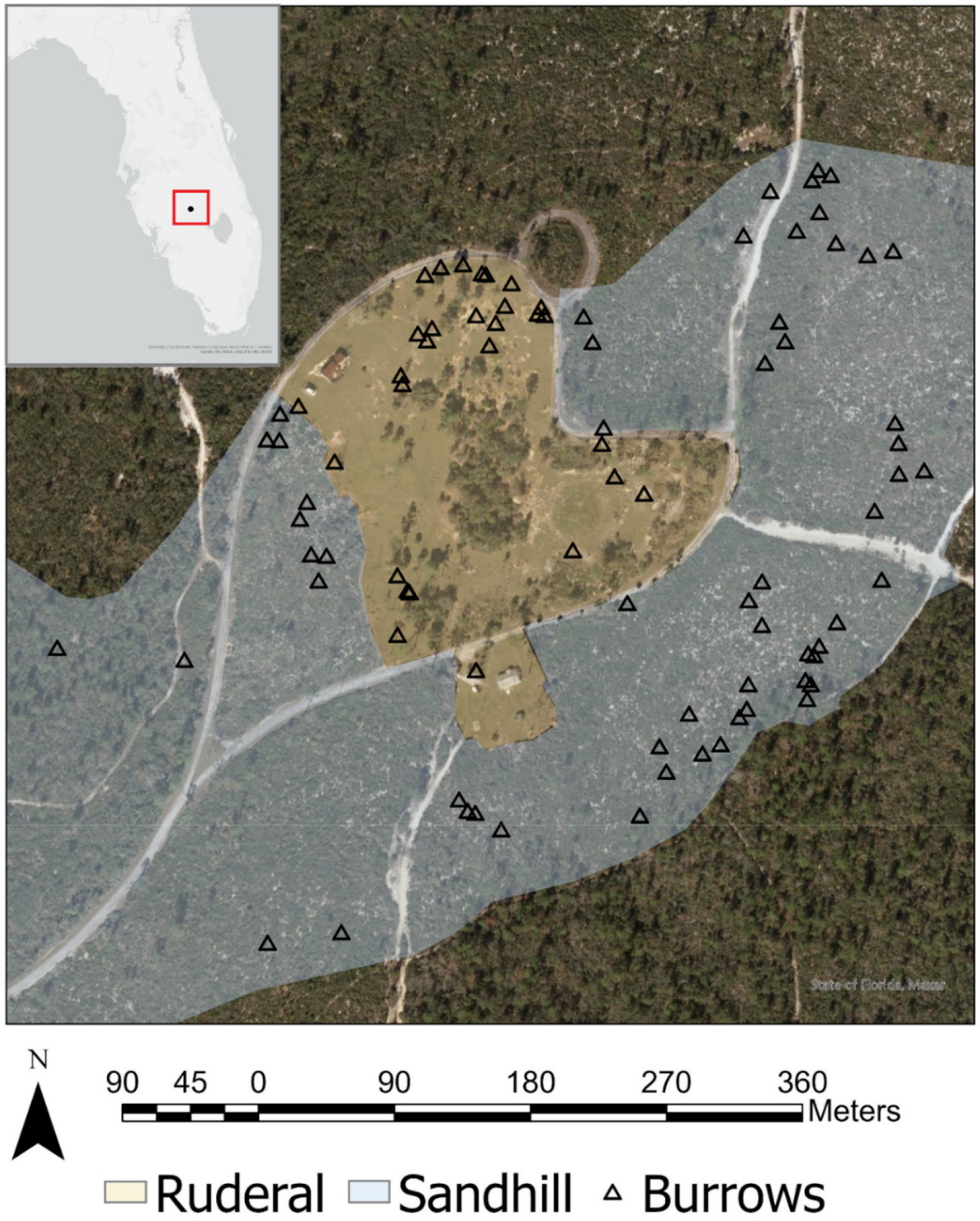
Aerial image of core study area in south‐central Florida showing locations of burrows (open triangles) and habitats used by radio‐tagged immature gopher tortoises from March 2021–September 2022. The areas most used by the tortoises are shaded in blue (fire‐maintained sandhill) and orange (ruderal). Much of the surrounding unshaded habitat is long‐unburned sandhill. Inset map in upper left shows location of study site in peninsular Florida.

We studied immature gopher tortoises in the “Red Hill” study population at Archbold Biological Station (Highlands Co., FL), which occupies 5 ha of ruderal habitat surrounded by approximately 84 ha of historically fire‐suppressed sandhill (wiregrass‐pine‐oak habitat; Figure 1). Ruderal habitat refers to vegetation communities growing on human‐disturbed sites, in this case an area that was used historically for agricultural purposes but is now dominated by non‐native grasses and kept open by mowing and occasional burning. Previous work has shown that adult tortoise densities in this habitat are higher than in adjacent sandhill (Howell et al. 2020). As of 2021, the adjacent sandhill units had a time‐since‐fire of 1–5 years, following 1–2 prescribed burns since 2013. Prescribed burns were typically preceded by mechanical clearing, i.e., mulching of sand pine (*Pinus clausa*), oaks (*Quercus* spp.), and other woody species, which aided reintroduction of fire and restoration of the desired open vegetation structure.

### 2.2 Tortoise Data Collection

**FIGURE 2:**
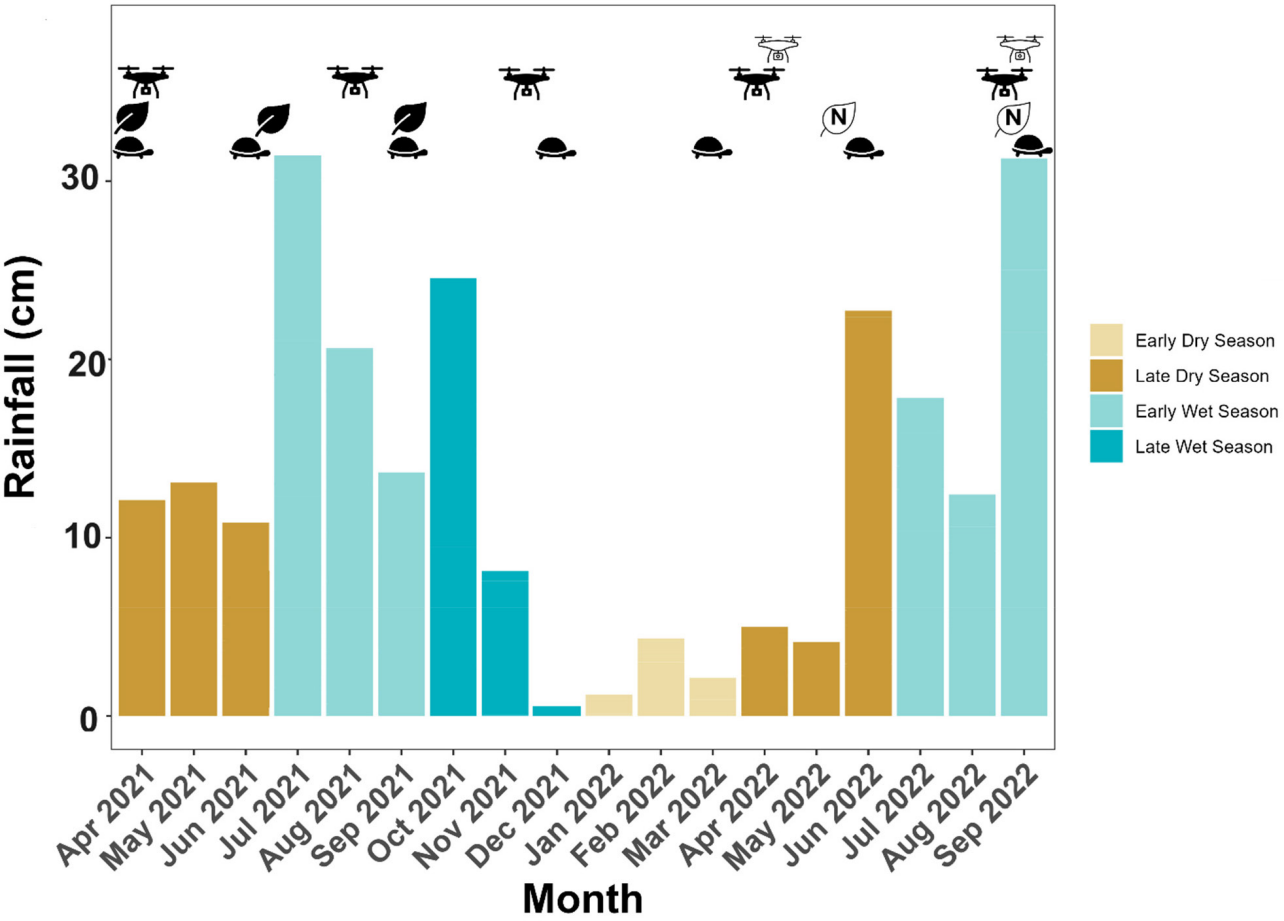
Monthly rainfall during each season and timing of gopher tortoise growth measurements (tortoise symbols), ground‐based plant surveys (black leaves), plant tissue collection for nutrient analysis (white leaves), RGB drone flights (black drones), and multispectral drone flights (white drones). Rainfall data were compiled from daily rainfall recorded manually at Archbold Biological Station's main weather station located 1 km from the study site.

Upon initial capture, each tortoise was measured, photographed, and given a unique set of marks by drilling or notching multiple marginal scutes. We captured tortoises by searching smaller burrows (≤ 24 cm wide) mapped during annual line‐transect surveys of the study area. To enable monitoring of individual habitat use and growth, we initiated capture and radio tracking of immature tortoises (1–5 years old) in Spring 2021. During the initial capture session, we tagged 10 tortoises in sandhill habitat and eight in ruderal habitat. In subsequent capture sessions, we tagged additional tortoises to bolster sample sizes and replace those lost to mortality or transmitter expiration. In total, we tagged 32 tortoises and tracked them two times per week for up to six seasons (mean 22.5 tortoises/season; Table A1). We deployed Advanced Telemetry Systems radio transmitters weighing 0.75 g (model R1635), 1.20 g (R1655), or 3.60 g (R1680), depending on size of the tortoise (range 68–1320 g, 68–175 mm initial carapace length). We recaptured tortoises quarterly for comprehensive morphometric measurements starting March 2021 until September 2022. For purposes of this study, we divided the year into early dry season (December to March), late dry season (March to June), early wet season (June to September), and late wet season (September to December; Figure 2). Morphometric measurements included carapace length (CL) and width, plastron length and width, and mass. Tortoise age was determined by counting the number of major annuli on the plastral scutes (Mushinsky, Wilson, and McCoy 1994).

### 2.3 Habitat Data Collection

#### 2.3.1 Forage Sampling

For the first three seasons of the study, we determined plant community composition by sampling 42 1‐m^2^ plots within 14 m of each burrow used by the radio‐tagged tortoises. Seven plots were spaced at 2‐m intervals along each of six transects radiating at 60° intervals from the burrow. We used 14 m as a radius because this is a realistic daily foraging distance for immature gopher tortoises (Wilson, Mushinsky, and McCoy 1994; Stemle, Rothermel, and Searcy 2022). We conducted a new vegetation survey whenever a tortoise moved to a new burrow (provided it stayed > 1–2 weeks) and once every season at burrows that were used for multiple seasons. Variables calculated from these surveys included frequency of non‐native bahiagrass (*Paspalum notatum*), native wiregrass (*Aristida stricta*), sedges, other non‐leguminous herbaceous species, native legumes, non‐native legumes, and tall woody vegetation (Table 1). Frequency was calculated as the proportion of the 42 plots surrounding each burrow in which the plant type was present.

**TABLE 1:** Predictor variables included in models of immature tortoise growth, and seasons when each variable was measured.

Variable name	Method	Description	2021			2022	
			Mar–Jun	Jun–Sep	Sep–Dec	Mar–Jun	Jun–Sep
BAHIAGRASS	GV	Frequency of non‐native bahiagrass	•	•	•		
WIREGRASS	GV	Frequency of native wiregrass	•	•	•		
OTHER HERB	GV	Frequency of other herbs	•	•	•		
LEGUME	GV	Frequency of native legumes	•	•	•		
NN LEGUME	GV	Frequency of non‐native legumes	•	•	•		
SEDGE	GV	Frequency of sedges	•	•	•		
TALL WOODY	GV	Frequency of tall woody plants	•	•	•		
GREEN HERB	D‐SC	% cover of green herbaceous veg	•	•	•	•	•
BROWN HERB	D‐SC	% cover of brown herbaceous veg	•	•	•	•	•
WOODY VEG	D‐SC	% cover of woody vegetation	•	•	•	•	•
TREE	D‐SC	% cover of trees	•	•	•	•	•
BARE GROUND	D‐SC	% cover of bare ground	•	•	•	•	•
MGRVI	D‐RGB	Modified Green Red VI	•	•	•	•	•
GLI	D‐RGB	Green Leaf Index	•	•	•	•	•
NDVI	D‐MS	Normalized Difference VI				•	•
NDRE	D‐MS	Normalized Difference Red Edge Index				•	•
EVI2	D‐MS	2‐band Enhanced VI				•	•
SRre	D‐MS	Red‐Edge Simple Ratio				•	•
CIgreen	D‐MS	Green Chlorophyll Index				•	•
CIred‐edge	D‐MS	Red Edge Chlorophyll Index				•	•
MTCI	D‐MS	MERIS Terrestrial Chlorophyll Index				•	•
RTVICore	D‐MS	Red‐Edge Triangulated VI				•	•

*Note:* All variables were measured within 14 m of burrows used by radio‐tagged tortoises. If a tortoise used two burrows within the same season, we calculated a weighted average of each metric based on the proportion of time the tortoise used each burrow.

Abbreviations: D‐MS = calculated from multi‐spectral drone data; D‐RGB = calculated from RGB drone data; D‐SC = supervised classification of RGB drone imagery; GV = ground‐based vegetation survey; VI = vegetation index.

During the late dry season (May) and early wet season (August) of 2022, we collected plant tissues to determine forage quality, i.e., nitrogen content. We sampled the three most abundant forage species in each of three randomly placed 1‐m^2^ plots within a 14‐m buffer surrounding the burrow of each radio‐tagged tortoise. The plot locations were chosen using random numbers generated for distance (1–14 m) and bearing (0°–360°) from the burrow. We collected enough leaves from each dominant species to reach ~2 mg of dry weight. In most cases, this meant collecting nearly all the leaves from multiple plants; however, we did not collect buds, flowers, or seeds. We placed the samples in paper bags and dried them in an oven at 50°C for 72 h, then sent the dried samples to the University of Georgia's Stable Isotope Lab for processing to determine total nitrogen (N) and carbon:nitrogen ratios.

#### 2.3.2 Supervised Classification of Drone Imagery

**FIGURE 3:**
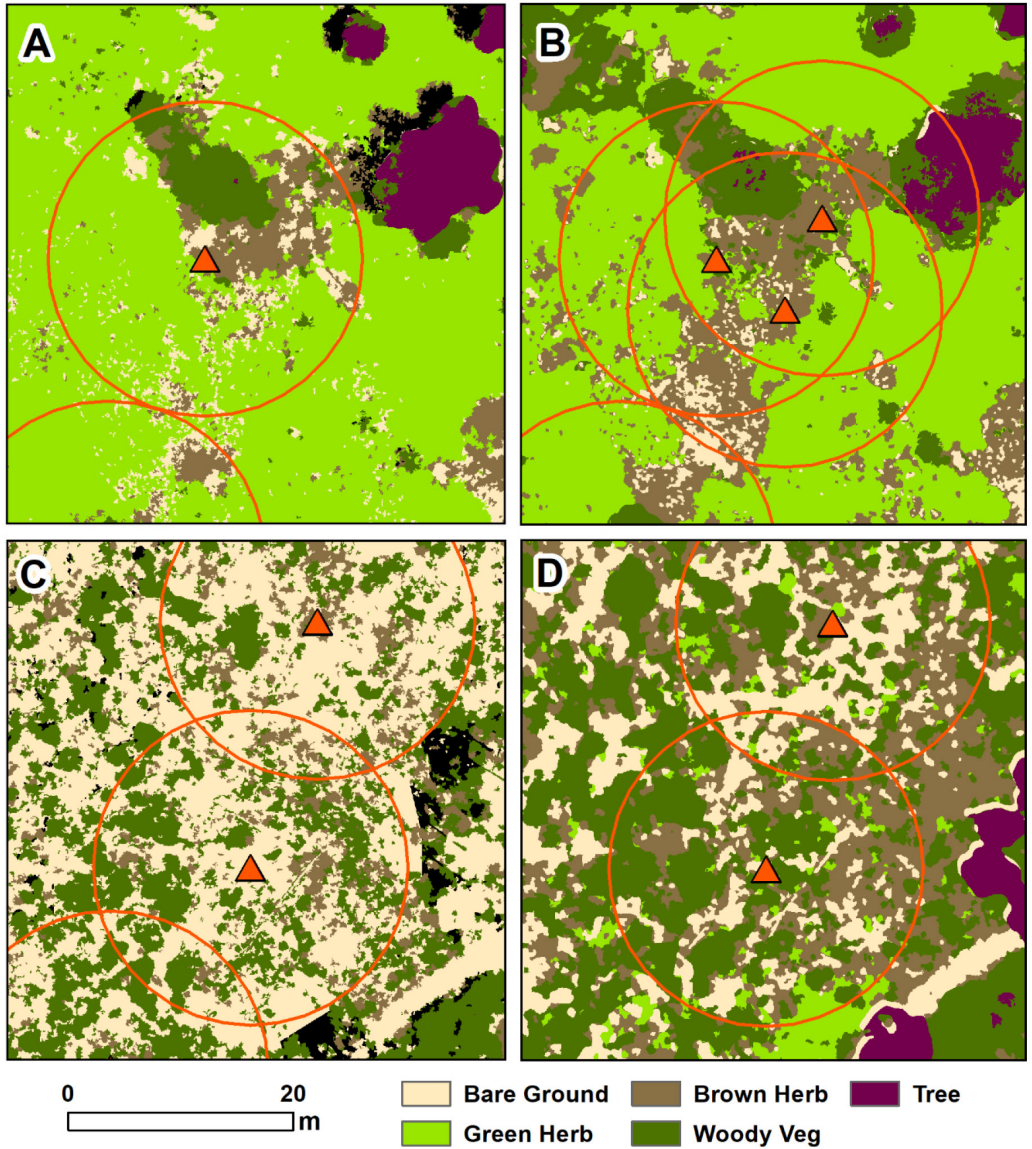
Examples of supervised classification of RGB drone imagery to characterize vegetation composition within 14 m of burrows used by radio‐tagged immature tortoises (orange triangles) in 2021. Panels A and B show an area of ruderal habitat in late dry season (left) and early wet season (right). Panels C and D show an area of native sandhill in late dry season (left) and early wet season (right).

We acquired high‐resolution Red‐Green‐Blue (RGB) aerial images (3.5‐cm resolution) with a DJI Phantom 4 Pro of the study area during five seasons (Figure 2), excluding the winter season (December 2021–March 2022) when there was negligible tortoise growth. Because of cloudy conditions in the late wet season of 2022, we had to conduct two drone flights (on 12 and 20 September) to obtain shadow‐free images of every burrow that were high enough quality for classification. The RGB images were post‐processed using ESRI Drone2Map to produce georectified orthomosaics for each date. In addition to this initial post‐processing, we used photogrammetry to produce a digital surface model (DSM) showing the elevation of the tallest object in each pixel and a digital terrain model (DTM), which categorizes the resulting point cloud and interpolates ground elevation values. We derived a vegetation height layer by subtracting the DTM from the DSM. To improve the accuracy of the classification, we used multiple subclasses to maximize spectral separation and then grouped those values into the final classes (e.g., we classified white sand and yellow sand separately, then merged these into one bare ground class). At the completion of the classification, we used the vegetation height layer to help correct obviously erroneously classified pixels (e.g., pixels classified as “tree” with a height value of < 1 m were reclassified to woody vegetation). To further improve the accuracy of our classification, we manually edited shadow pixels within the burrow buffers to estimate which classes were not visible because of shadows. We then employed supervised classification in ArcGIS Pro (v 2.9, ESRI, Redlands, CA) to determine percent cover of bare ground, brown herb, green herb, trees, and woody vegetation within 14 m of each burrow (Figure 3).

#### 2.3.3 Calculation of Vegetation Indices

We calculated two vegetation indices (VIs) from RGB drone imagery for all five seasons. Modified Green Red Vegetation Index (MGRVI) and Green Leaf Index (GLI) were calculated with the *raster calculator* tool in ArcGIS Pro and the mean, max, and 90th percentile were extracted from the 14‐m buffers with the *zonal statistics* tool. In addition to the RGB imagery, we acquired multispectral imagery in May and September 2022 using a DJI Matrice 300 RTK quadcopter equipped with a MicaSense RedEdge‐MX sensor and downwelling light sensor. The flights occurred within 2 h of solar noon at a height of 120 m AGL, yielding a resolution of ~10 cm. The resulting imagery was processed in Agisoft Metashape Professional Edition to obtain radiometrically calibrated orthomosaics consisting of blue, green, red, red edge, and near‐infrared bands. These orthomosaics were then used to calculate eight multispectral VIs using the *raster* package in R version 4.2.1 (R Core Team 2022) similar to Walsh et al. (2018). These multispectral VIs were loaded as rasters into ArcGIS Pro and georeferenced to the RGB imagery for each season. The multispectral VIs were then extracted to the 14‐m buffers around each burrow using the *zonal statistics as table* tool in ArcGIS Pro to get the maximum, mean, and 90th percentile within the buffers.

### 2.4 Statistical Analyses

#### 2.4.1 Growth Analyses

For tortoises tracked at least 12 months (March 2021–March 2022), we used a two‐sample t‐test to compare annual growth (CL % growth per day) between habitats. For individuals tracked all six seasons (see Table A1), we ran a repeated‐measures analysis of variance (ANOVA) to examine effects of habitat, time (i.e., season), and their interaction on growth measured at quarterly intervals. The repeated‐measures ANOVA was conducted in R Studio using the *dplyr* package (Wickham et al. 2022). Post hoc tests consisted of one‐way ANOVAs comparing growth between habitats within each season using the Benjamini‐Hochberg procedure to correct for multiple comparisons.

To evaluate which variables were the best predictors of tortoise growth (CL % growth per day), we utilized model selection using the *dredge* function in R Studio (Bartoń 2022) to identify the best‐supported model based on AICc. We compared each set of predictors and their *R* ^2^‐values for each season, as different subsets of predictors were available for each season (Table 1). We included initial CL as a covariate in all models to account for ontogenetic change in growth rate. To ensure no variables were correlated in each top model, we verified that all Variance Inflation Factors were < 5.

#### 2.4.2 Forage Analyses

We used a factorial ANOVA to examine effects of season and habitat (ruderal versus sandhill) on the N content of forage species averaged across the three plots at each burrow. Additionally, we compared percent total N between habitats for each season separately using one‐way ANOVAs.

#### 2.4.3 Survival Analyses

Using a POPAN formulation of the Jolly‐Seber model in package *marked* (R Studio v 4.0.0, R Core Team 2022; Laake, Johnson, and Conn 2013), we estimated the quarterly apparent survival of the radio‐tracked tortoises from March 2021 to September 2022. We considered models with both constant and time‐varying values for apparent survival (phi) and recapture probability (pent), resulting in four candidate models. We used the model with the lowest AICc to estimate survival of all immature tortoises, as well as small (< 120 mm CL) versus large (> 126 mm CL) tortoises separately. In addition, we estimated apparent survival separately for each habitat, excluding three tortoises that spent significant amounts of time in both habitats (see Table A1). For habitat‐specific analysis, we only included tortoises that spent > 80% of their time in a single habitat during the 18‐month study period.

## 3 Results

### 3.1 Tortoise Growth

#### 3.1.1 Seasonal and Habitat‐Specific Growth Rates

**FIGURE 4:**
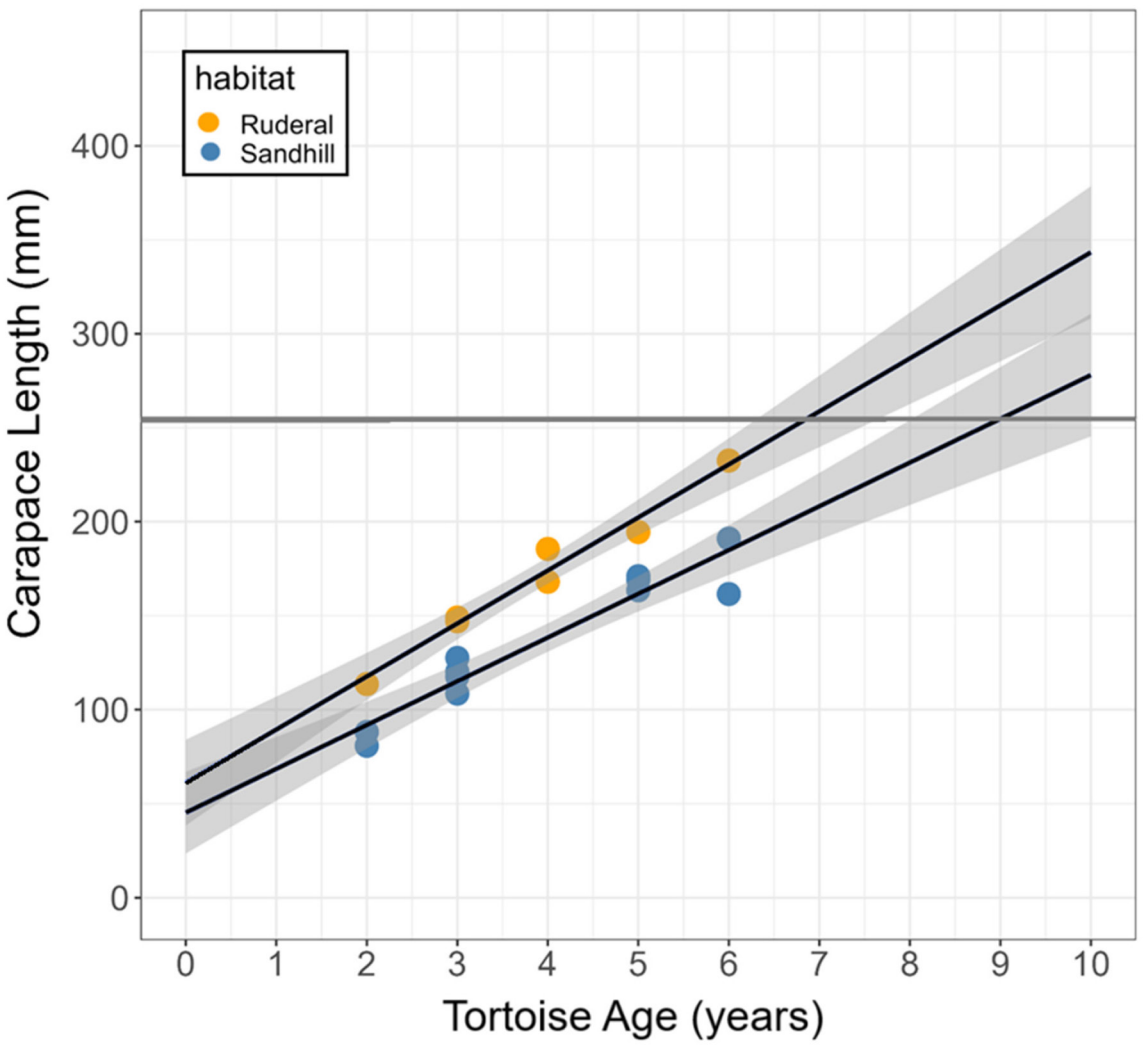
Relationships between body size and age for immature gopher tortoises in ruderal (*n* = 7) and sandhill (*n* = 11) habitats, with 95% confidence bands. Age was based on number of annuli counted when collecting the last measurements of each tortoise during the study. Based on linear projections, tortoises in ruderal habitat are predicted to reach the minimum female size at maturity (horizontal gray line; Meshaka Jr., Layne, and Rice 2019) significantly earlier (~2 years earlier) than tortoises in sandhill.

**FIGURE 5:**
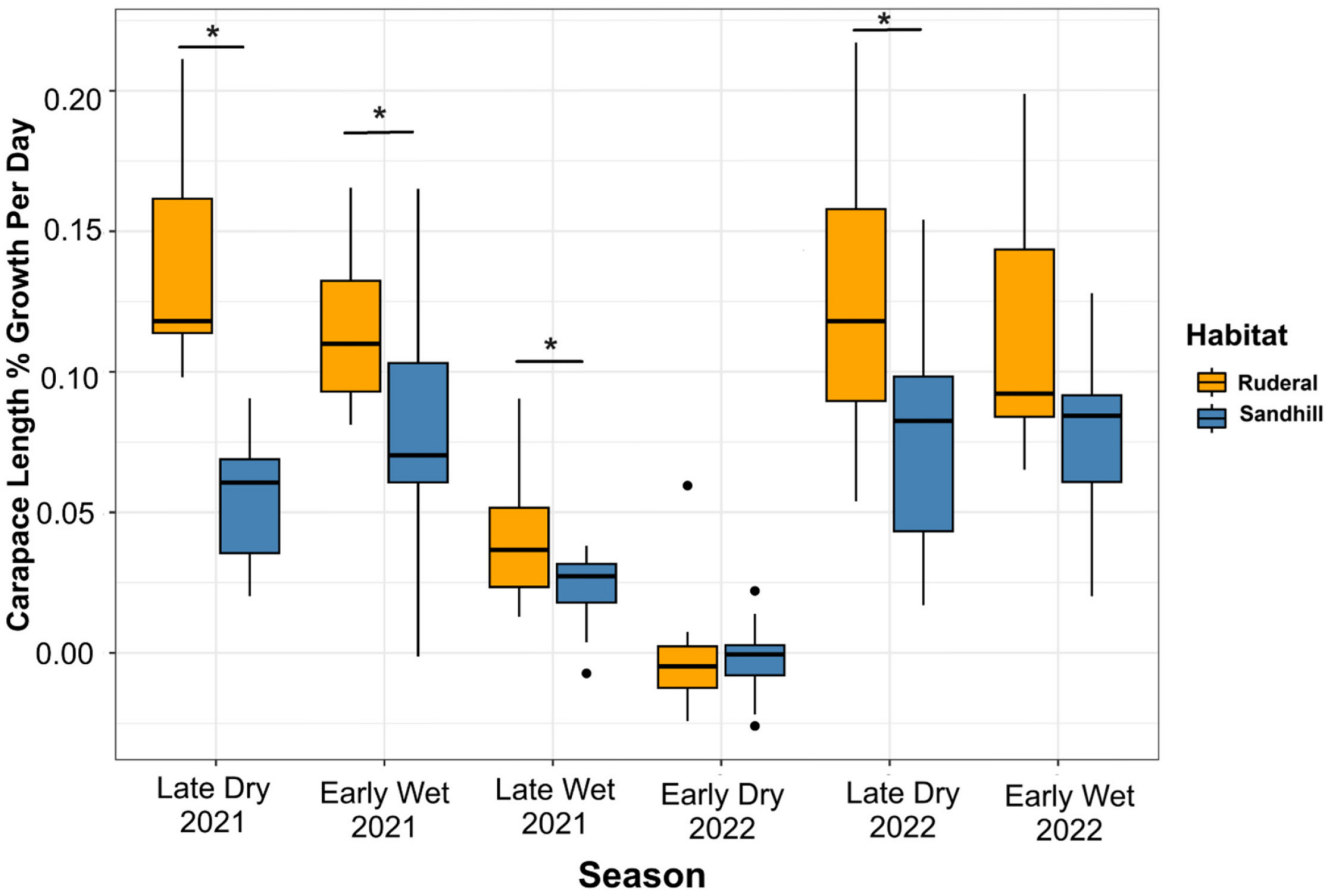
Mean percent growth in carapace length per day in each habitat and season. *p < 0.05. Overall, growth was higher in ruderal habitat (*p* = 0.003), although this growth advantage varied seasonally (*p* < 0.001), with the greatest advantage observed in the late dry season (Mar–June).

From March 2021 to March 2022, tortoise growth rate differed significantly between ruderal and sandhill habitat (*F*_1,13_ = 12.0, *p* = 0.004; Figure 4). Mean annual growth rates were 34.4 ± 3.5 mm (*n* = 6) in ruderal habitat and 19.5 ± 3.4 mm (*n* = 8) in sandhill. Quarterly growth rates differed significantly between habitats (*F*_1,10_ = 6.9, *p* = 0.003) and across seasons (*F*_5,50_ = 40.4, *p* < 0.001; Figure 5, Table A2), including a significant habitat x season interaction (*F*_5,50_ = 4.4, *p* < 0.001; Figure 5). Growth was consistently higher in ruderal habitat, with the strongest effect in the late dry season and smaller effects in the early wet and late wet seasons (Tables A2 and A3). There was no difference in growth between habitats in the early dry season, because growth was near zero during this coldest part of the year. Growth also did not vary significantly between habitats during the early wet season of 2022, which may be due to the lower sample size compared to the early wet season of 2021 (Tables A2 and A3).

#### 3.1.2 Predictors of Tortoise Growth

**FIGURE 6:**
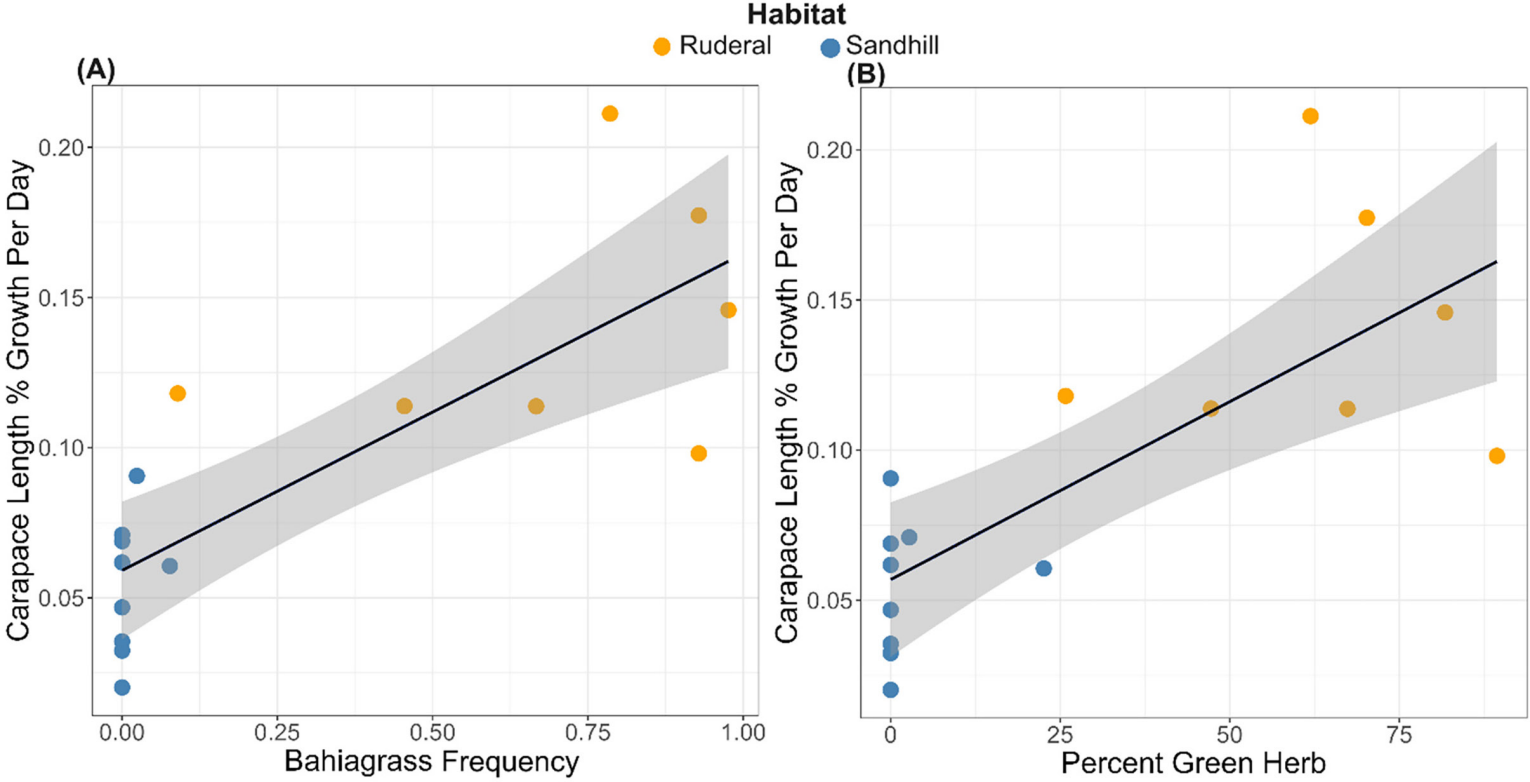
Top predictors of immature gopher tortoise growth during late dry season of 2021. Frequency of non‐native bahiagrass (A) was the strongest predictor of growth from ground‐based plant surveys, whereas percent cover of green herb (B) was the strongest drone‐derived predictor.

When available, data from ground‐based plant surveys were always more predictive of tortoise growth than remotely acquired data. We found positive effects of BAHIAGRASS, SEDGE, and NN LEGUME on growth and negative effects of TALL WOODY (Table 2). The environmental variables were most predictive of tortoise growth in the late dry season of 2021 (Figure 6), during which the drone‐derived indices explained a similar percentage of the variation in immature tortoise growth (*R* ^2^ = 0.64) as the ground‐based plant surveys (*R* ^2^ = 0.71). The drone flight in April 2022 corresponded with a period of much lower rainfall compared to April 2021 (Figure 2), and our models explained very little of the variation in growth during late dry season of 2022. Instead, in 2022 our models were most predictive of growth during the early wet season, after the onset of rain alleviated the seasonal drought. Among the drone‐derived indices, GREEN HERB was the best predictor of tortoise growth (top model in three of five seasons; Table 2; Figure 6). WOODY VEG, which is largely the inverse of GREEN HERB (*r* = −0.89, *p* < 0.001), was also revealing. Two of the VIs based on RGB data provided useful drone‐derived predictors of immature tortoise growth. Specifically, GLI was negatively correlated with tortoise growth, while MGRVI was positively associated. GLI measures density of dark green wavelengths (oak leaves have high value), while MGRVI discriminates between healthy plants (high value) and bare soil/ground (low value). In contrast, none of the VIs based on multispectral data were good predictors of growth (Table 2).

**TABLE 2:** Best models of tortoise growth by season (see Table 1 for variable descriptions and seasons measured).

Season	Ground‐based plant survey	Drone—supervised classification	Drone—RGB indices	Drone—multispectral indices
Late dry 2021 (Mar–Jun)	*R* ^2^ = 0.71 **BAHIAGRASS** (0.839) Initial CL (−0.153)	*R* ^2^ = 0.64 **GREEN HERB** (0.798) Initial CL (−0.211)	*R* ^2^ = 0.43 **90% GLI** (−0.590) Initial CL (−0.000)	NA
Early wet 2021 (Jun–Sep)	*R* ^2^ = 0.55 **SEDGE** (0.516) **NN LEGUME** (0.651) **Initial CL** (−0.367)	*R* ^2^ = 0.28 **GREEN HERB** (0.429) Initial CL (−0.310)	*R* ^2^ = 0.23 MGRVI Max (0.372) Initial CL (−0.279)	NA
Late wet 2021 (Sep–Dec)	*R* ^2^ = 0.43 SEDGE (0.358) **TALL WOODY** (−0.370) Initial CL (−0.320).	*R* ^2^ = 0.24 **GREEN HERB** (0.410) Initial CL (−0.363)	Null model	NA
Late dry 2022 (Mar–Jun)	NA	*R* ^2^ = 0.31 **WOODY VEG** (−0.438) Initial CL (−0.252)	*R* ^2^ = 0.27 MGRVI Max (0.392) Initial CL (−0.336)	Null model
Early wet 2022 (Jun–Sep)	NA	*R* ^2^ = 0.44 WOODY VEG (−0.371) **Initial CL** (−0.544)	*R* ^2^ = 0.65 **MGRVI Max** (0.844) **90% GLI** (−0.739) **Initial CL** (−0.426)	*R* ^2^ = 0.65 **NDVI Max** (0.436) **90% NDVI** (−0.422) **Initial CL** (−0.613)

*Note:* Values in parentheses are standardized regression coefficients and significant relationships (*p* < 0.05) are indicated in bold. Early dry season is not included due to negligible growth during this period.

### 3.2 Forage Composition and Quality

Based on ground‐based plant surveys, bahiagrass was the dominant forage plant in ruderal habitat (42.8% of plots), whereas wiregrass was dominant in sandhill (45.5% of plots; Tables A4 and A5). Thus, the difference in forage quality between the two habitat types can largely be attributed to these two species. Wiregrass consistently had the lowest N content (mean 0.66%) and bahiagrass consistently had higher N (~2‐fold) than wiregrass (two‐sample t‐test, *p* < 0.001; Table A5). However, the difference in forage quality between the two habitat types decreased between the late dry and early wet seasons for two reasons. First, N content of bahiagrass decreased significantly (by 21%) over this time interval (two‐sample t‐test, *p* < 0.001), diminishing N availability in the ruderal habitat. Second, the diversity of forage species in sandhill increased (late dry season Shannon diversity = 2.40; early wet season Shannon diversity = 3.69), decreasing the influence of the N‐poor wiregrass. Legume frequency increased in both habitats from May to August, but the increase in sandhill was much larger (199% increase) than the increase in ruderal habitat (75%). In both seasons, leguminous (native or non‐native) species had higher N than other species (May: *F*_1,15_ = 6.3, *p* = 0.024; August: *F*_1,18_ = 7.2, *p* = 0.015), driving their importance for forage quality.

Collectively, the seasonal and habitat‐specific patterns of N content of forage species mirrored the observed patterns of immature tortoise growth. Forage species in ruderal habitat had significantly higher total N than forage in sandhill habitat in the 2022 late dry season (*F*_1,84_ = 6.1, *p* = 0.015), when tortoise growth was similarly greater in ruderal habitat (Figure 5). Total N did not differ between habitats in the 2022 early wet season (*F*_1,133_ = 0.1, *p* = 0.770), when there was no statistically significant difference in growth between the two habitats (Table A3).

### 3.3 Tortoise Survival

For all time periods and analyses, the top model had constant survival across time periods (phi ~ 1). Quarterly apparent survival of tagged immature tortoises across both habitats from March 2021–September 2022 was 94.2% ± 2.2 (95% CI: 88.0%–97.3%; *n* = 32). Quarterly apparent survival was similar for immature tortoises residing in ruderal habitat (94.9% ± 3.5, 95% CI: 82.1%–98.7%; *n* = 12) and in sandhill (93.1% ± 3.3, 95% CI: 83.0%–97.4%; *n* = 16). These estimates correspond to annual apparent survival rates of 81.1% and 75.1% for ruderal and sandhill habitats, respectively, or 79.3% for all tortoises combined. Quarterly apparent survival did not differ significantly between size classes, although point estimates were lower for small tortoises (91.6% ± 3.5, 95% CI: 81.6%–96.4%; *n* = 19) than for large tortoises (96.4% ± 2.5, 95% CI: 86.8%–99.1%; *n* = 13). These estimates correspond to annual apparent survival rates of 70.4% (tortoises < 120 mm CL) and 86.4% (tortoises > 126 mm CL).

### 3.4 Time Investment per Method

Drone imagery required a ~0.5‐h flight if it was for a RGB‐derived vegetation index or ~2 h for a multispectral index, and many hours of post‐processing and computer run time; the estimated total time for calculating spectral indices was approximately 30 h, with multispectral taking closer to 35 h (25–40 h). Supervised classification required a ~0.5‐h drone flight, ~8 h classification time, and many hours of post‐processing and computer run time; the estimated total time for supervised classification was approximately 38 h. Plant surveys required ~60 person‐hr of fieldwork (30–60 min per burrow with 2–4 people) plus 5 h entering data, for a total estimate of 65 h per season. Thus, considering it took ~65 h for plant surveys and ~34 h for drone data acquisition (averaging 30 h for spectral indices and 38 h for supervised classification), drone‐based methods required ~48% less time.

## 4 Discussion

Our examination of individual variation in growth rates of immature gopher tortoises with respect to forage resources provides valuable new insights for wildlife managers aiming to maintain population resilience under anthropogenically modified disturbance regimes. Although our 1.5‐year study only spanned two growing seasons, we found consistent patterns in immature gopher tortoise growth, which varied significantly by both habitat and season. In ruderal habitat, we observed the highest annual growth rates ever reported for wild gopher tortoises, an average of 34.4 mm/year compared with 5–19 mm/year at other sites throughout the species' range (Harris 2014). In comparison, immature tortoises in sandhill habitat grew only slightly faster than those at another sandhill site in central Florida (19.5 mm/year vs. 19 mm/year; Mushinsky, Wilson, and McCoy 1994). Within our study population, there is a strong positive relationship between immature tortoise growth and abundance of bahiagrass and other non‐native grasses/legumes (found mostly in ruderal habitats), whereas growth is negatively correlated with abundance of woody vegetation (found mostly in sandhill). These patterns were apparent not only from ground‐based plant surveys but also using supervised classification or vegetation indices derived from high‐resolution drone imagery (see Table 2), enabling similar estimates of forage quality over much larger areas in the future. We further demonstrate that the advantage in forage quality of the ruderal habitat is most prevalent during the late dry season, a time when native food resources are scarce. Additionally, we have developed a mechanistic understanding of growth rate variation by finding an identical habitat‐by‐season pattern in the N content of available forage. In support of this conclusion, previous work has shown N‐rich plants are important for growing gopher tortoises (Mushinsky, Stilson, and McCoy 2003; Hathaway 2012).

Our study also clarifies the effects of habitat quality on demography by demonstrating how habitat heterogeneity contributes to within‐population differences in age‐specific body size. Specifically, our linear model of size versus age projects that tortoises in ruderal habitat at Archbold Biological Station can attain the minimum size for sexual maturity ~2 years earlier than those in sandhill habitat (Figure 4), and likely earlier than reported for any other population (Harris 2014; Meshaka Jr., Layne, and Rice 2019). Rapid growth enables young tortoises to reach sexual maturity faster, thus increasing fitness by reducing time at a vulnerable size and expanding the number of reproductive years (Mangel and Stamps 2001; Harris 2014). Just as fine‐scale differences in habitat quality create divergent growth trajectories within a population, site‐to‐site differences in habitat quality combined with latitudinal variation in length of the activity season, result in widely varying mean growth rates among populations throughout the species' range (Mushinsky et al. 2006).

Our hypothesis of a growth‐survival trade‐off was not supported because apparent survival estimates showed no evidence of negative effects of ruderal habitat on survival of this age class (1–7 years). If anything, survival tended to be higher in the ruderal habitat and thus in the same direction as that habitat's higher growth potential. This contrasts with previous hypotheses that faster growth could be associated with trade‐offs in fitness, such as increased predation risk while foraging or reduced immune function (Lindeman 1997; Mangel and Stamps 2001), which still need to be examined. Generally, mowed grass habitats are considered an ecological trap for turtle species, as they are often associated with roadsides, where turtles face higher mortality risk (Aresco 2005; Shepard et al. 2008; Rautsaw et al. 2018). However, at our study site, there is high driver awareness of tortoises and mowing is restricted to early morning when tortoises are less active aboveground. Additionally, survivorship could differ among sites due to differences in predator community composition or differences in vegetation structure and cover (e.g., grass height, and shrub density). Thoroughly addressing the question of growth‐fitness trade‐offs in human‐altered habitats will require data from additional sites and measuring fitness traits over longer periods, because mortality could be higher in response to more extreme weather events or unique combinations of environmental stressors not captured in this relatively short study. Ideally, future studies should also include neonates, as it remains unclear if gopher tortoises would experience a growth‐survival trade‐off during the first year post‐hatching.

Given ongoing urbanization and projected population declines (USFWS 2021; Folt et al. 2022; Loope, Akçakaya, and Shoemaker 2024), we expect more intensive management interventions will be required to stabilize gopher tortoise populations. Mitigation translocations of gopher tortoises are already common (Seigel and Dodd Jr. 2000), particularly in Florida, where thousands of permits have been issued in recent years for onsite relocation or offsite translocation of tortoises from areas slated for development to permitted recipient sites with suitable habitat (USFWS 2021). Both translocated and natural populations may be subject to increased predation rates, either from subsidization of mesopredators (Harden, Price, and Dorcas 2009) or spread of novel invasive predators (Offner, Campbell, and Johnson 2021). These rising threats lend urgency to research on factors influencing recruitment and ways to enhance juvenile growth and survival to boost population resiliency. In the presence of greater predation pressure, promoting faster juvenile growth might improve demographic resilience by reducing the window of extreme vulnerability (Folt et al. 2021). Along these lines, our results point to intentional manipulation of forage resources as a useful avenue for future research.

As we have shown, the availability, abundance, and higher N content of non‐native grasses and other plants during the late dry season and into the early wet season enhance tortoise growth in ruderal habitat. Thus, interspersion or maintenance of some ruderal habitat could be beneficial to gopher tortoises in certain situations. For example, habitat management options are often limited in urbanizing landscapes due to restrictions on prescribed burning (Yager et al. 2007; USFWS 2021; Folt et al. 2022). We suggest it would be worthwhile to test the demographic effects of establishing small, interspersed plots of perennial, non‐native forage, particularly in areas where native groundcover is lacking or difficult to establish. Plants that have high forage value, such as legumes, could also be incorporated. While forage supplementation alone might offer limited benefits, it could be a useful, low‐cost tool when combined with other intensive management efforts, for example, to enhance post‐release growth and site fidelity of head‐started or translocated tortoises (Tuberville, Quinn, and Buhlmann 2021; McKee et al. 2021). Manipulation of forage availability and cover is a common management practice for quail and other game birds (Brunk et al. 2023), though supplemental feeding has had varied success (Henry et al. 2022). Importantly, we are not advocating conversion of high‐quality native habitat to artificially created “food plots” to support gopher tortoises, as this would be a species‐specific viewpoint that fails to consider the conservation value of the entire sandhill community. It is also critical to avoid introduction or facilitation of invasive predators (e.g., red imported fire ants; *Solenopsis invicta*), or invasive plants, but bahiagrass has not shown a tendency to invade sandhill at our study site (Rothermel, pers. obs). In general, we see a need to investigate this and other novel habitat management techniques that might benefit this keystone species.

Use of drone imagery is becoming prevalent in ecological and conservation research (Blake et al. 2013; Jiménez López and Mulero‐Pázmány 2019; Marcelino et al. 2020). Although ground‐based plant surveys always provided the strongest predictors of immature tortoise growth in our study, drone data were able to closely approximate the same result in the late dry/early wet season when plant growth is stimulated by increased precipitation. During this time of year, the drone data identified green herbaceous cover (or GLI, MGVRI, NDVI) as the primary driver of immature tortoise growth, which encompasses the more refined driver of bahiagrass identified by ground‐based plant surveys. Moreover, while both conventional field methods and drone data provided weaker predictors of growth during the remaining seasons, our repeated‐measures analysis indicated that ruderal habitat supported the fastest growth throughout the entire year, and it was just the magnitude of this advantage that varied seasonally, not the direction of the effect. Thus, identifying the best growth habitat using a drone flight in the late dry/early wet season when the predictive power is highest would not lead one to identify habitat that was suboptimal at other times of year. However, given differences in rainfall and vegetation phenology throughout the range of gopher tortoises, the optimal timing of flights would likely vary as well. For areas north of central Florida, if there is some supplemental forage available during the driest part of the active season (when temperatures are high enough for foraging), then we would expect to see similar effects on growth. A similar study conducted in the northern part of the species' range could clarify.

In summary, our results generated an improved mechanistic understanding of juvenile growth, which informs lifetime fitness. As the enhanced growth of immature (1‐ to 7‐year‐old) tortoises does not trade off with lower survival, well‐timed drone imagery can be used to identify high‐quality habitat. Although drone‐based habitat assessment requires access to a drone and sufficient data storage capability, as well as significant post‐processing time, we found a roughly 50% time savings compared to ground‐based surveys. We therefore suggest that supervised classification or vegetation spectral indices (not necessarily multispectral indices) of high‐resolution drone imagery are useful methods for assessing gopher tortoise forage resources. We especially see a role for drones when there is a need to assess large areas, such as entire preserves or recipient sites, as these remote methods scale up more efficiently than ground‐based plant surveys. Thus, our study adds another example to a growing list of potential applications of drone technology that can facilitate more effective, cost‐efficient habitat monitoring and management to benefit imperiled species (Baratchi et al. 2013).

## Author Contributions

**Leyna R. Stemle:** conceptualization (equal), data curation (supporting), formal analysis (lead), funding acquisition (equal), investigation (equal), methodology (equal), project administration (equal), software (equal), supervision (equal), validation (equal), visualization (lead), writing – original draft (lead), writing – review and editing (lead). **Julie M. Sorfleet:** data curation (equal), formal analysis (equal), methodology (equal), software (equal), writing – review and editing (supporting). **Chelsea L. Moore:** data curation (equal), investigation (equal), writing – original draft (supporting), writing – review and editing (supporting). **Jack T. Christie:** data curation (equal), investigation (equal), software (equal), visualization (supporting), writing – original draft (supporting), writing – review and editing (supporting). **Christopher A. Searcy:** conceptualization (equal), data curation (supporting), formal analysis (supporting), funding acquisition (supporting), investigation (supporting), methodology (supporting), resources (supporting), supervision (supporting), validation (supporting), writing – original draft (supporting), writing – review and editing (lead). **Betsie B. Rothermel:** conceptualization (equal), data curation (equal), formal analysis (supporting), funding acquisition (lead), investigation (lead), methodology (equal), project administration (lead), resources (lead), supervision (lead), validation (equal), visualization (supporting), writing – original draft (supporting), writing – review and editing (lead).

## Conflicts of Interest

The authors declare no conflicts of interest.

## Data Availability

Data and code for the project can be found on GitHub: https://github.com/lstemle/Immature_GT_growth_survival.git.

## Appendix A

**TABLE A1:** Body size, age, tracking history, and seasonal habitat assignments for every gopher tortoise included in the study of immature growth and survival at Archbold Biological Station, Florida, in 2021–2022.

Tortoise ID	Initial CL (mm)	No. annuli (age) in 2021	No. annuli (age) in 2022	Season 1 Mar–Jun 21	Season 2 Jun–Sep 21	Season 3 Sep–Dec 21	Season 4 Dec 21–Mar 22	Season 5 Mar–Jun 22	Season 6 Jun–Sep 22
1300	99.0	3	4	**R**	**R**	**R**	**R**	**R**	**R**
1559	176.0	5	6	**R**	**R**	**R**	**R**	**R**	**R**
1690	68.2	2	3	**R**	**R**	**R**	**R**	**R**	**R**
1691	128.1	3	4	**R**	**R**	**R**	**R**	**R**	**R**
1720	128.4	4	5	**R**	**R**	**R**	**R**	**R**	**R**
1730	173.5	4	5	**R**	R/S	R/S	**S**	R/S	**S**
1620	136.6	3	—	**R**	**R**	**R**	R/S	Unknown fate (went missing after 4/14/22)	—
1380	90.4	2	—	**R**	Unknown fate (went missing after 6/3/21)	—	—	—	—
1820	72.9	1	2	—	**R**	**R**	**R**	**R**	**R**
1670	114.0	2	3	—	**R**	**R**	**R**	**R**	Found dead 7/22/22
1612	110.5	2	3	—	**R**	**R**	Known alive (tag died 12/16/21)	Known alive	Known alive (recap 9/16/22)
1630	89.7	2	—	—	**R**	**R**	Known alive (tag died 11/27/21)	Known alive	Known alive (recap 9/6/23)
1703	80.1	2	3	—	**R**	**R**	Known alive (tag died 11/19/21)	Known alive	Known alive (recap 9/16/22)
1711	142.0	3	4	—	**R**	**R**	**S**	**R**	R/S
1681	116.1	3	4	—	—	—	—	**R**	**R**
1328	87.3	2	3	**S**	**S**	**S**	**S**	**S**	**S**
1370	127.5	3–4	4–5	**S**	**S**	**S**	**S**	**S***	**S**
1640	156.0	4–5	5–6	**S**	**S**	**S**	**S**	**S**	**S**
1650	80.6	2	3	**S**	**S**	**S**	**S**	**S***	**S**
1740	132.0	5	6	**S**	**S**	**S**	**S**	**S***	**S***
1760	133.5	4	5	**S**	**S**	**S**	**S**	**S**	**S**
1790	126.5	3–4	4–5	**S**	**S**	**S**	**S**	**S**	**S**
1780	118.4	3	—	**S**	**S**	**S**	**S**	Unknown fate (lost tag, went missing late May)	—
1750	157.0	4	—	**S**	Unknown fate (went missing after 6/10/21)	—	—	—	—
1770	75.5	1–2	—	**S**	Unknown fate (lost tag, went missing late Aug)	—	—	—	—
1696	65.0	1	2	—	**S**	**S**	**S**	**S**	**S**
1830	91.4	2	3	—	**S**	**S**	**S**	**S**	**S**
1840	94.8	2	3	—	**S**	**S**	**S**	**S**	**S**
1697	117.0	3	4	—	**S**	R/S	R/S	R/S	**R**
1704	81.3	2	—	—	**S**	**S**	Unknown fate (tag died 11/19/21)	—	—
1712	73.2	—	2	—	—	—	—	**S**	**S**
1170	156.5	—	5	—	—	—	—	—	**S**

*Note:* Seasons included in the growth analysis are in bold. *Habitat codes*: R = spent at least 80% of its time in ruderal. S = spent at least 80% of its time in sandhill. R/S = spent significant time in both habitats. *dispersed out of the core study area during this season but stayed in native habitat. *Notes about fate*: “Known alive” = not tracked to end of study but known alive because individual was recaptured during subsequent searches of burrows in the study area. Others remained “unknown fate” because they were never recaptured after their tags died or fell off.

**TABLE A2:** Seasonal mean percent growth in carapace length (CL % growth per day) in each habitat, based on every radio‐tagged tortoise we obtained measurements for in that season (provided they could be classified as living in a single habitat type).

Season	Ruderal		Sandhill	
	*N*	Mean (*SD*)	*N*	Mean (*SD*)
Late dry 2021 (Mar–Jun)	7	0.140% (0.00039)	9	0.054% (0.00022)
Early wet 2021 (Jun–Sep)	12	0.116% (0.00038)	13	0.077% (0.00045)
Late wet 2021 (Sep–Dec)	12	0.041% (0.00024)	12	0.023% (0.00014)
Early dry 2021–2022 (Dec–Mar)	7	0.001% (0.00027)	13	−0.003% (0.00014)
Late dry 2022 (Mar–Jun)	9	0.120% (0.00050)	11	0.070% (0.00041)
Early wet 2022 (Jun–Sep)	7	0.120% (0.00052)	13	0.103% (0.00040)

**TABLE A3:** Results of separate one‐way ANOVAs testing for effects of habitat (ruderal vs. sandhill) on immature tortoise growth in each season.

Season	*F*	*df* (*N* − 2)	*p*
Late dry 2021 (Mar–Jun)	28.7	14	< 0.001*
Early wet 2021 (Jun–Sep)	6.7	23	0.017*
Late wet 2021 (Sep–Dec)	4.9	22	0.038*
Early dry 2021–2022 (Dec–Mar)	0.2	18	0.631
Late dry 2022 (Mar–Jun)	6.2	18	0.023*
Early wet 2022 (Jun–Sep)	3.9	18	0.064

*Note: p*‐Values were corrected for multiple comparisons using the Benjamini‐Hochberg procedure.

* Significant.

**TABLE A4:** Frequency of different plant taxa within 14 m of burrows used by immature gopher tortoises in ruderal and sandhill habitats at Archbold Biological Station, Florida.

Plant taxon	Ruderal			Sandhill		
	Late dry	Early wet	Late wet	Late dry	Early wet	Late wet
Wiregrass	0.02	0.00	0.01	0.40	0.34	0.40
Other native grass	0.18	0.13	0.15	0.45	0.46	0.39
Bahiagrass	0.69	0.68	0.65	0.02	0.02	0.01
Other nonnative grass	0.43	0.53	0.59	0.15	0.25	0.29
Native legume	0.32	0.26	0.16	0.53	0.58	0.36
Nonnative legume	0.13	0.29	0.36	0.04	0.03	0.02
Other herbaceous	0.67	0.63	0.54	0.44	0.50	0.52
Sedge	0.10	0.18	0.11	0.07	0.20	0.06
Tall woody shrub	0.01	0.06	0.06	0.27	0.32	0.27

*Note:* Vegetation surveys occurred in 2021 during the late dry season (12 May–7 June; *n* = 23 burrows), early wet season (6 August–27 September; *n* = 36), and late wet season (8 November–4 January; *n* = 31). Frequency was calculated as the mean proportion of plots across all burrows sampled containing each type of plant, averaged across all burrows sampled within that season.

**TABLE A5:** Mean percent total nitrogen (N) and mean carbon:nitrogen ratio (C:N) of forage species in late dry season (May) and early wet season (August) of 2022 in ruderal and sandhill habitat at Archbold Biological Station, Florida.

Plant species	Mean % Total N (*SD*)				Mean C:N (*SD*)			
	Ruderal		Sandhill		Ruderal		Sandhill	
	May	Aug	May	Aug	May	Aug	May	Aug
*Andropogon* spp. Bluestem	—	—	1.03 (0.03) *N* = 2	1.14 (0.26) *N* = 3	—	—	44.84 (1.93) *N* = 2	40.34 (8.98) *N* = 3
*Aristida stricta* Wiregrass	—	0.73 *N* = 1	0.68 (0.10) *N* = 20	0.63 (0.19) *N* = 20	—	61.42 *N* = 1	67.20 (10.09) *N* = 20	76.67 (18.69) *N* = 20
*Schizachyrium* sp. Bluestem	—	—	0.81 (0.06) *N* = 2	—	—	—	55.24 (3.96) *N* = 2	—
*Imperata cylindrica* **Cogongrass**	1.44 *N* = 1	—	—	—	31.80 *N* = 1	—	—	—
*Melinis repens* **Natal grass**	0.89 (0.19) *N* = 4	0.87 (0.28) *N* = 7	—	1.49 (0.25) *N* = 2	51.34 (11.79) *N* = 4	55.59 (20.50) *N* = 7	—	28.63 (4.15) *N* = 2
*Paspalum notatum* **Bahiagrass**	1.57 (0.22) *N* = 18	1.24 (0.25) *N* = 24	—	—	28.18 (3.63) *N* = 18	35.84 (7.16) *N* = 24	—	—
*Sporobolus indicus* **Smutgrass**	0.93 (0.15) *N* = 3	0.69 (0.24) *N* = 7	0.85 *N* = 1	1.46 (0.05) *N* = 3	47.80 (7.64) *N* = 3	68.89 (24.61) *N* = 7	50.30 *N* = 1	29.77 (1.39) *N* = 3
*Chamaecrista fasciculata* Partridge pea	—	—	—	2.71 (0.54) *N* = 7	—	—	—	18.99 (4.12) *N* = 7
*Chapmannia floridana* Florida alicia	—	—	2.20 (0.07) *N* = 2	1.91 (0.35) *N* = 6	—	—	20.35 (0.87) *N* = 2	23.32 (4.12) *N* = 6
*Galactia elliottii* Elliott's milkpea	2.01 (0.14) *N* = 5	2.10 (0.09) *N* = 6	2.32 *N* = 1	—	23.76 (1.55) *N* = 5	22.18 (1.09) *N* = 6	19.12 *N* = 1	—
*Galactia regularis* Eastern milkpea	—	2.92 *N* = 1	—	—	—	14.74 *N* = 1	—	—
*Rhynchosia cinerea* Brownhair snoutbean	—	—	—	2.60 *N* = 1	—	—	18.25 *N* = 1	—
*Tephrosia* sp. Hoarypea	—	—	2.45 (0.09) *N* = 6	2.00 (0.50) *N* = 12	—	—	19.31 (0.78) *N* = 6	27.63 (19.45) *N* = 12
*Grona triflora* **Ticktrefoil**	2.27 (0.31) *N* = 2	2.44 (0.20) *N* = 6	—	—	19.83 (2.52) *N* = 2	18.61 (1.75) *N* = 6	—	—
*Indigofera hirsuta* **Hairy indigo**	—	3.52 *N* = 1	—	—	—	12.01 *N* = 1	—	—
*Cleome* sp. **Spiderflower**	—	4.14 *N* = 1	—	—	—	9.68 *N* = 1	—	—
*Erigeron canadensis* Canadian horseweed	2.59 *N* = 1	—	—	—	15.88 *N* = 1	—	—	—
*Geobalanus oblongifolius* Gopher apple	1.24 (0.16) *N* = 3	1.15 (0.20) *N* = 2	1.26 (0.04) *N* = 3	1.16 (0.19) *N* = 3	37.33 (5.23) *N* = 3	39.58 (6.46) *N* = 2	36.94 (1.44) *N* = 3	39.00 (5.61) *N* = 3
*Pityopsis graminifolia* Narrowleaf silkgrass	1.28 (0.13) *N* = 2	1.22 (0.24) *N* = 5	1.50 (0.19) *N* = 4	1.37 (0.23) *N* = 6	32.80 (2.66) *N* = 2	37.51 (8.42) *N* = 5	29.11 (3.15) *N* = 4	32.74 (4.95) *N* = 6
*Richardia* sp. **Mexican clover**	1.75 *N* = 1	1.74 *N* = 1	—	—	23.26 *N* = 1	21.01 *N* = 1	—	—
Unknown mint Lamiaceae	1.83 *N* = 1	2.35 *N* = 1	—	—	24.14 *N* = 1	18.54 *N* = 1	—	—
*Vitis rotundifolia* Muscadine	1.74 *N* = 1	—	1.66 (0.29) *N* = 3	1.69 (0.40) *N* = 7	25.37 *N* = 1	—	26.89 (4.58) *N* = 3	27.96 (8.42) *N* = 7

*Note:* Dashes (–) indicate no plants of that species were sampled during that month. Non‐native species are in bold. Standard deviations are in parentheses.
